# The Lifespan-Promoting Effect of Otophylloside B in *Caenorhabditis elegans*

**DOI:** 10.1007/s13659-015-0064-4

**Published:** 2015-06-26

**Authors:** Jie Yang, Qin-Li Wan, Quan-Zhang Mu, Chun-Feng Wu, Ai-Jun Ding, Zhong-Lin Yang, Ming-Hua Qiu, Huai-Rong Luo

**Affiliations:** State Key Laboratory of Phytochemistry and Plant Resources in West China, Kunming Institute of Botany, Chinese Academy of Sciences, 134 Lanhei Road, Kunming, 650201 Yunnan China; University of Chinese Academy of Sciences, Beijing, 100049 China; The Second Affiliated Hospital of Kunming Medical University, Kunming, 650101 China

**Keywords:** Otophylloside B, *Caenorhabditis elegans*, Aging, DAF-16/FOXO, IIS signaling pathway

## Abstract

Aging is the major risk factor for many human diseases and degeneration. Thus, clinically effective medicine could delay the process of aging and aging-related diseases are desperately wanted. In traditional Chinese medicine (TCM), some were claimed to slow down aging. Qingyangshen (*Cynanchum otophyllum* schneid) is such a TCM. Here, we assayed the longevity effect of compound Otophylloside B (Ot B), a C-21 steroidal glycoside isolated from Qingyangshen, in *Caenorhabditis elegans*, which is a popular model for aging research. Our results showed that Ot B could modestly extend the lifespan of *C. elegans*, delay the age-related decline of body movement and improve the stress resistance. Further investigating the molecular mechanism of lifespan extension effect revealed that Ot B could activate the FOXO transcription factor DAF-16. Ot B could not further extend the lifespan of long-lived mutant of insulin/IGF-1-like receptor (*daf*-*2*). In addition, Ot B also requires SIR-2.1 and CLK-1 which is an enzyme in ubiquinone synthesis, for lifespan extension.

**Electronic supplementary material:**

The online version of this article (doi:10.1007/s13659-015-0064-4) contains supplementary material, which is available to authorized users.

## Introduction

The identification of chemical interventions that can ameliorate age-related illness and degeneration has been an important aspect of current aging research. Pharmacological compounds that could slow down the normal aging process and extend the lifespan, could also delay the progression of age-related disorders, such as Alzheimer’s disease and cardiovascular diseases. In addition, the characterizing the mechanism of the lifespan extension effect of drugs can help to understand endogenous mechanisms involved in longevity. The genetic pathways identified to regulate longevity were turned out to be evolutionarily conserved. For example, the insulin/IGF-1-like signaling (IIS) [1–3] is highly conserved to influence longevity in model organisms ranging from worms to mice [4].

The free-living soil nematode *C. elegans* has been a leading system for studying genetic and pharmacologic influences on lifespan, mainly because of its short lifespan and amenability to genetic manipulation. In addition, a number of compounds have been reported to extend *C. elegans* lifespan, such as a variety of antioxidant compounds [5, 6], complex mixtures derived from plants [7, 8], a sirtuin activator resveratrol [9, 10], an antihyperglycemic drug metformin [11, 12], TOR inhibition rapamycin [13], nonsteroidal anti-inflammatory drugs (NSAIDs) (e.g. aspirin), as well as anticonvulsant medicines (e.g. ethosuximide) [14, 15].

Otophylloside B (Ot B), a C-21 steroidal glycoside, was isolated from *C. otophyllum* (Chinese name Qingyangshen) [16], which was a folk medicinal plant endemic to Yunnan province of China, whose root was used for the treatment of epilepsy, rheumatic pain, kidney weakness, and muscle injuries by the local people in its growing area. However, the molecular mechanism activated by Ot B remains vague. In our study, we found that Ot B could lengthen the lifespan of *C. elegans*, delay the age-related decline of body movement and improve heat resistance. We further demonstrated that Ot B-mediated lifespan extension depend on the FOXO transcription factor DAF-16. The effect of Ot B on lifespan could share similar phenotypic features as IIS signaling pathway because it failed to further extend the lifespan of long-live insulin-like receptor mutant *daf*-*2* [17]. In addition, Ot B extending the lifespan also requires SIR-2.1 and CLK-1.

## Results and Discussion

### Ot B Extended the Lifespan of *C. elegans*, Delayed Age-Related Decline of Body Movement, and Increased Heat Stress-Resistance

**Fig. 1:**
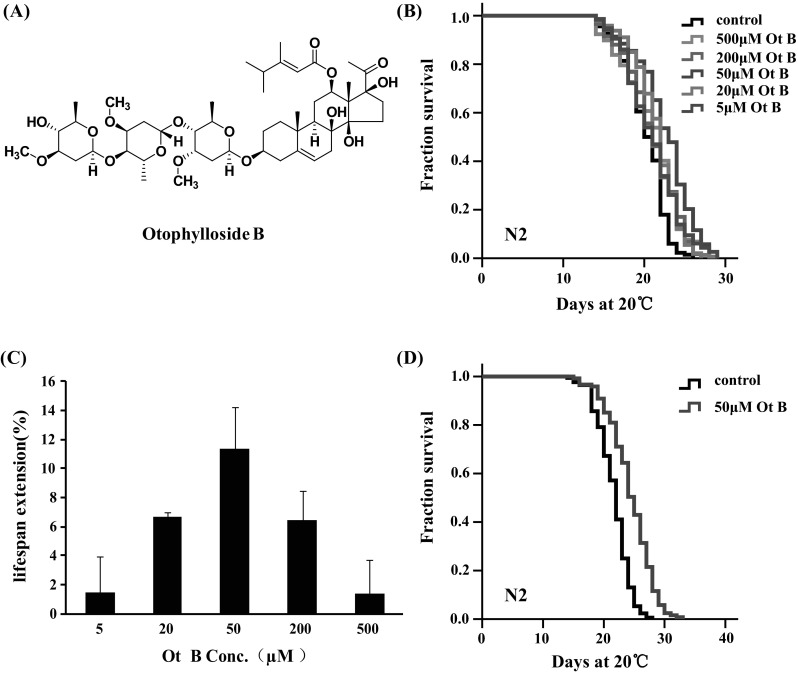
Otophylloside (Ot B) extended adult lifespan in *C. elegans*. **a** Chemical structure of Ot B. **b** Survival curves of wild-type (N2) animals raised at 20 °C on nematode growth media plates containing either no Ot B or different concentrations of Ot B(5, 20, 50, 200, and 500 μM). **c** Doseage–response analysis of Ot B. Wild-type (N2) animals was treated with 5, 20, 50, 200, and 500 μM Ot B. The average percentage change in lifespan of at least two independent experiments was plotted as a function of dosage. **d** Survival curves of wild-type (N2) animals raised on 0 (*black*) and 50 μM (*red*) Ot B plates at 20 °C. All experiments the treatments were initiated from the first day of adulthood and continued until death. Statistical details and repetition of this experiment are summarized in Table S1 (Supplementary information)

One goal of aging study is to identify drugs that can slow aging and delay age-related illness and degeneration. To identify compounds that might slow aging and extend lifespan in *C. elegans*, we assayed a panel of compounds with pharmacological effects or bioactivity related to improving health condition or reducing the age-related diseases, such as qingyangshengenin, otophylloside A, otophylloside B and *β*-sitosterol, which were isolated from *C. otophyllum*. Among those examined, we found that Ot B (Fig. 1a) could extend the lifespan of *C. elegans*. Dose–response analyses indicated that 50 μM of Ot B displayed the largest lifespan extension by up to 11.3 % (Fig. 1c, d). Animals exposed to either higher or lower than 50 μM of Ot B exhibited a smaller or an insignificant lifespan extension (Fig. 1b, c).

**Fig. 2:**
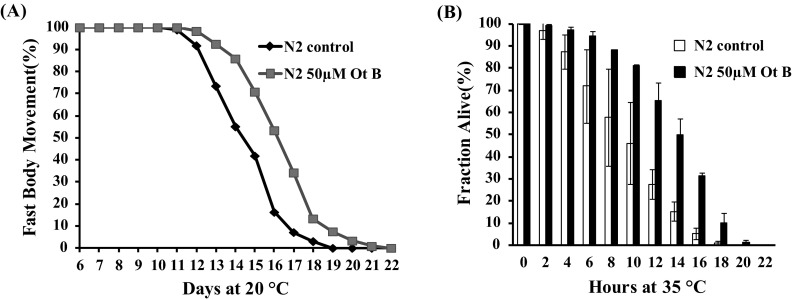
Otophylloside (Ot B) delayed age-associated body movement changes and improved thermotolerance. **a** Age-related movement of worms non-treated and treated with 50 μM Ot B. The movement was assorted as fast movement and not fast movement. The mean fast body movement span was identified as a phenotype of age, Table S3 (supplementary information). **b** The survival percentage of WT worms cultured at 35 °C non-treated and treated with 50 μM Ot B. The* figures* showed the mean lifespan of at least three independent experiments, and* error bars* represented standard deviation (SD). In each experiment, the *P* values were calculated by K–M method log rank test and *P* < 0.001. The statistical details are summarized in the Table S2 (Supplementary information)

The body movement of *C. elegans* is the one of most obvious behavioral abnormality accompanying nematode aging [18]. We investigated if Ot B could delay the age-related decline of body movement. In both treated and non-treated animals, body movement declined progressively during aging. However, Ot B treated animals exhibited significantly lower decline of body movement than non-treated controls (Fig. 2a).

*Caenorhabditis elegans* with extended lifespan often presents increased stress resistance [19]. So we examined the effect of Ot B on the lethality of heat stress. As shown in Fig. 2b, Ot B treatment suppressed the lethality of heat stress in wild-type *C. elegans*.

Altogether, by a combination of assays of lifespan, body movement, and heat stress, we showed the anti-aging effect of otophylloside B in *C. elegans.*

### Ot B Extends the Lifespan of *C. elegans* Through FOXO Transcription Factor DAF-16

**Fig. 3:**
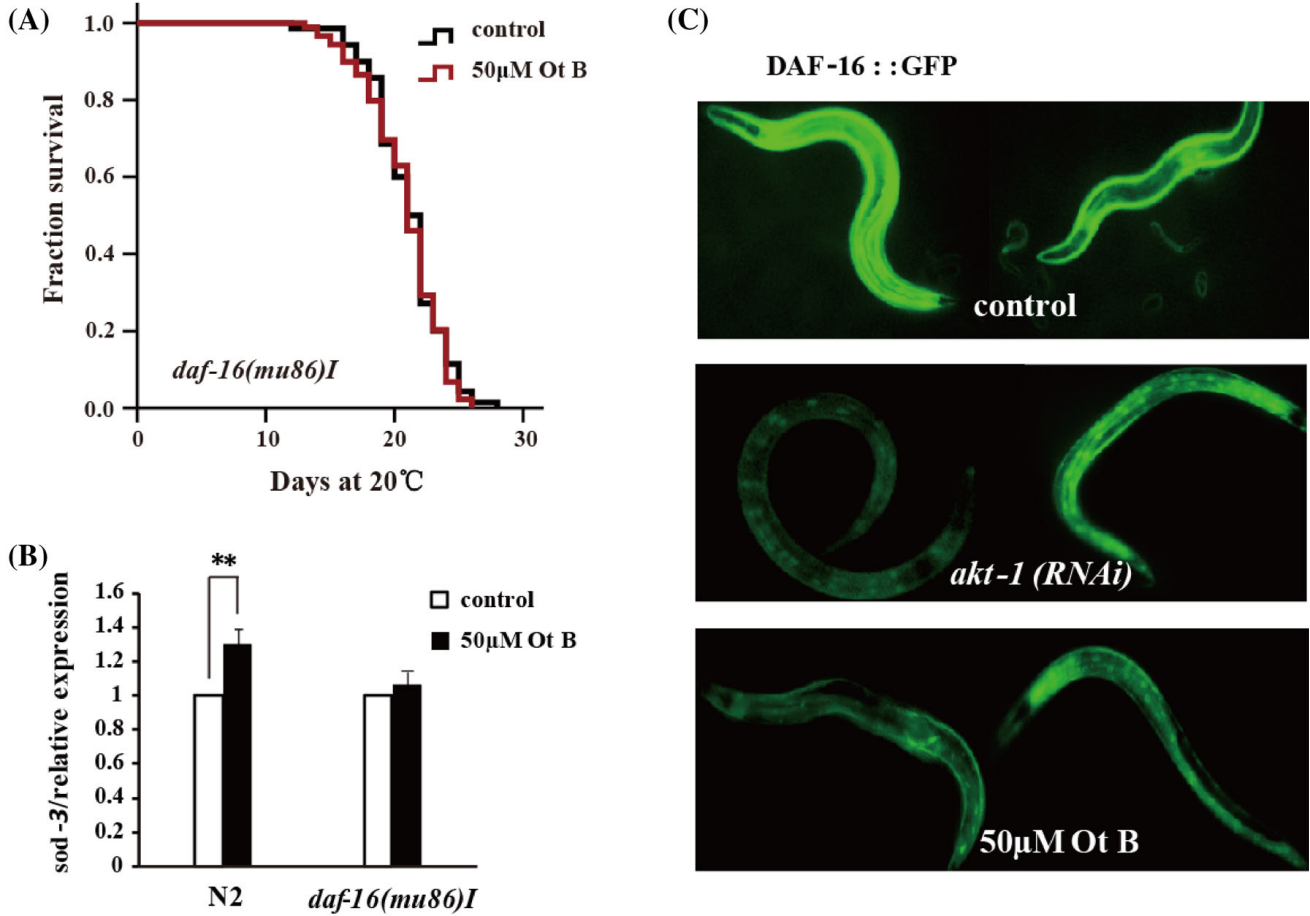
Otophylloside (Ot B) extended adult lifespan in a DAF-16-dependent manner. **a** Survival curves of *daf*-*16* mutants grown on 0 (*black*) and 50 μM (*red*) Ot B plates at 20 °C, Ot B could not further extend mean lifespan of daf-16 mutants. In all cases, these data represent the results of a single trial. Repetition of this experiment and statistical details are summarized in Table S1 (supplementary information). **b** *sod*-*3* mRNA was increased 1.4-fold in treated with 50 μM Ot B worms compared with non-treated worms. The results presented correspond to the mean and SEM of two independent experiments: **P = 0.007643 in* t* test. **c** Ot B could cause DAF-16 nuclear localization. DAF16::GFP-expressing worms were placed on 0 and 50 μM Ot B plates at 20 °C for 24 h. RNA interference treatment by feeding of *akt*-*1* was performed as a positive control

*Caenorhabditis elegans* DAF-16, the homolog of mammalian Forkhead box O transcription factors (FOXO), was crucial in mediating stress resistance and longevity [2, 17, 20]. We tested if DAF-16 played a role in lifespan extension by Ot B. Our result showed that Ot B could not extend the lifespan of *daf*-*16* null mutant *daf*-*16(mu86) I* [21] (Fig. 3a).

To further examine that Ot B did act on DAF-16, we examined the effect of Ot B on the expression of *sod*-*3* by qRT-PCR, which is a known DAF-16 target gene involved in both stress resistance and longevity [22, 23]. The expression of *sod*-*3* was significantly increased when wild type N2 worms exposed in 50 μM of Ot B for 24 h (Fig. 3b). Besides, Ot B could not increase the expression of *sod*-*3* in *daf*-*16* null mutant exposed in Ot B for 24 h (Fig. 3b). These results suggested Ot B-induced lifespan extension depended on DAF-16.

Previous studies have shown that DAF-16 accumulates in the nucleus when the activity of its upstream kinases is reduced [2]. Thus, we examined whether Ot B could trigger the nuclear localization of DAF-16. In agreement with our model, we found Ot B increased the level of nuclear-localized DAF-16::GFP fusion after 24 h of treatment with Ot B (Fig. 3c), suggesting Ot B treatment might promote DAF-16 activation.

### Effect of Ot B on Lifespan Extension may be Conferred by a Reduction in Insulin/IGF-1-Like Signaling

**Fig. 4:**
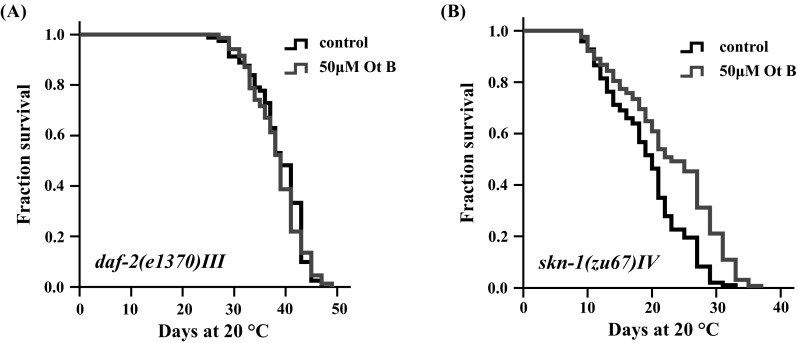
Lifespan extension by Otophylloside (Ot B) was mediated by IIS signaling pathway. Survival curves of **a** *daf*-*2(e1370) III*, **b** *skn*-*1(zu67) IV* grown on plates containing 0 (*black*) and 50 μM (*red*) Ot B at 20 °C. Ot B could not further extend the lifespan of *daf*-*2(e1370) III*, but could extend the lifespan of *skn*-*1(zu67) IV*. All drug treatments were initiated from the first day of adulthood and continued until death. In all cases, these data represent the results of a single trial. Repetition of this experiment and statistical details are summarized in Table S1 (supplementary information)

Daf-16 was known to receiving several upstream inputs to regulate their downstream effects, such as IIS pathway and the silent information regulator 2 (*SIR2*) [24]. To gain insight into the function of Ot B in lifespan extension, we examined the effect of Ot B on lifespan of the long-lived insulin-like receptor mutant *daf*-*2(e1370) III* [25]. Our results showed that Ot B could not lengthen the lifespan of *daf*-*2* mutant (Fig. 4a). Thus, effect of Ot B on lifespan extension might be conferred by insulin/IGF-1-like signaling.

While previous study indicated that IIS signaling inhibits the activation of DAF-16, as well as SKN-1, which was both functionally and structurally related to mammalian Nrf transcription factor [26]. The *C. elegans* SKN-1 was shown to play a role in lifespan extension [26–28]. Thus, we investigated whether SKN-1 mediated the lifespan extension by Ot B. We found that Ot B could further increase the mean lifespan of *skn*-*1(zu67) IV* (Fig. 4b), suggesting that SKN-1 did not play a role in Ot B-induced lifespan extension.

### Ot B-Induced Lifespan Extension Requires SIR-2.1 but was Independent of *eat*-*2* Minic Dietary Restriction Mechanism

**Fig. 5:**
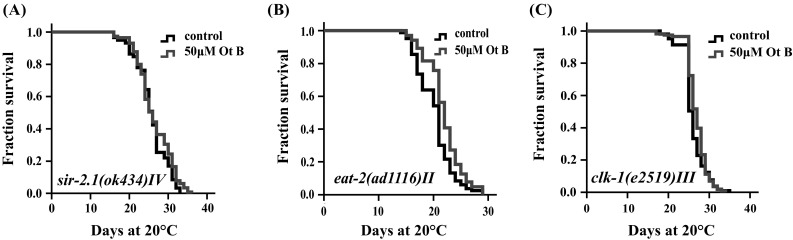
Otophylloside (Ot B)-related lifespan extension required for SIR-2.1 and reduction of mitochondrial respiration, but could not further extend the lifespan of *eat*-*2*. Survival curves of **a** *sir*-*2.1(ok434) IV*, **b** *eat*-*2(ad1116) II*, **c** *clk*-*1(e2519) III*. Ot B could not further extend mean lifespan of *sir*-*2.1(ok434) IV* and *clk*-*1(e2519) III,* but could further extend lifespan of *eat*-*2*. In all cases, these data represent the results of a single trial. Repetition of this experiment and statistical details are summarized in Table S1 (supplementary information)

The silent information regulator 2 (*SIR2*), a nicotinamide adenine dinucleotide (NAD)-dependent deacetylase, could bind to DAF-16 and extend the lifespan of *C. elegans* [29–31]. We investigated if Ot B could act on SIR-2.1 to extend the lifespan of *C. elegans* with a null mutant strain *sir*-*2.1(ok434) IV* [32]. Ot B could not increase the lifespan of *sir*-*2.1* mutant (Fig. 5a), indicating that SIR-2.1 is necessary for Ot B-mediated lifespan extension.

Previous studies have reported SIR-2.1 was a key mediator of the beneficial effects of dietary restriction, which is able to integrate sensing of the metabolic status with adaptive transcriptional outputs [33, 34]. The pharyngeal pumping defective mutant *eat*-*2(ad1116) II* was considered DR-constitutive for its reduced food intake and extended lifespan [35]. Treatment with 50 μM of Ot B at 20 °C could further increase the lifespan of *eat*-*2(ad1116) II*, suggesting that Ot B might not act through a *eat*-*2* minic DR mechanism (Fig. 5b).

### Ot B Might Extend Lifespan by Mediating to Reduce Mitochondrial Respiration

Oxidative stress is a crucial factor that influences aging. Mitochondria respiration plays a major role in reactive oxygen species production and has been reported to mediate aging process [35, 36]. The mutation of mitochondrial respiration components, such as *clk*-*1*, the enzyme in ubiquinone synthesis, was shown to reduce the oxygen consumption. The *clk*-*1* mutant *clk*-*1(e2519) III* has the defect of mitochondria respiration and were long-lived [36, 37]. Ot B treatment could not further extend the lifespan of mutants *clk*-*1(e2519) III*, indicating that Ot B might extend lifespan by mediating to reduce mitochondrial respiration (Fig. 5c).

In *C. elegans*, DAF-16 was the main effecter of the well-characterized IIS signaling pathway [22, 25]. Ot B promoted DAF-16 nuclear localization (Fig. 3c), and increased the expression level of DAF-16 regulated gene *sod*-*3*(Fig. 3b), and could not extend the lifespan of the *daf*-*16* null mutant *daf*-*16(mu86) I* (Fig. 3a). Above results suggested that Ot B might act in IIS signaling pathway to extend lifespan. This hypothesis was consistent with our results that Ot B could not further extend the lifespan of the long-lived *daf*-*2* mutant (Fig. 4a). But the lack of a negative effect of Ot B on the other mutants could not rule out the possibility that Ot B might act on other pathways upstream of DAF-16 [38]. We also found Ot B failed to increase the lifespan of *sir*-*2.1* (Fig. 5a) and *clk*-*1* (Fig. 5c). Therefore, these results suggested that Ot B-induced lifespan of *C. elegans* was not specific to reducing IIS activity, but also depended on other pathways, such as SIR-2.1 and mitochondrial respiration.

Based on activation effect of Ot B on DAF-16, here we considered that there are three possibilities to explain this finding. One possibility is that Ot B directly binds DAF-16 and promotes DAF-16 nuclear localization. The second possibility is that Ot B acts on the protein that functions upstream of DAF-16 in a linear pathway, such as *daf*-*2* or *age*-*1*, to activate DAF-16. The third possibility is that the effects of Ot B on DAF-16 indirectly acted on proteins whose function is far upstream of the IIS signaling pathway, and simultaneously acted on protein in parallel to the IIS signaling pathway to regulate DAF-16. To understand the specific mechanism that the Ot B activates DAF-16 to extend the lifespan, further work is required to confirm our expectation.

## Conclusion

Ot B, a C-21 steroidal glycoside, was isolated from C. otophyllum in 1986 and reported to have bioactivity of anti-epilepsy [16], but the molecular mechanism remains unclear. Our results suggest that 50 μM Ot B extend the lifespan of *C. elegans* by up to 11.3 %, delay the age-related decline of body movement and improve the stress resistance. Further investigating the molecular mechanism of lifespan extension effect revealed that Ot B could activate the FOXO transcription factor DAF-16 and Ot B could not further extend the lifespan of long-lived mutant of insulin/IGF-1-like receptor (*daf*-*2*). Besides, Ot B also requires SIR-2.1 and CLK-1 which is an enzyme in ubiquinone synthesis, for lifespan extension.

## General Experimental Procedures

### Chemicals and Strains

All stains were obtained from *Caenorhabditis* Genetics Center (CGC). The following strains were used in this study: wild type N2, DA1116 *eat*-*2(ad1116) II*, VC199 *sir*-*2.1(ok434) IV*, CB4876 *clk*-*1(e2519) III*, CB1370 *daf*-*2(e1370) III*, TJ356 *daf*-*16(zls356) IV*, CF1038 *daf*-*16(mu86) I,* and EU1 *skn*-*1(zu67) IV*. All strains grew and maintained on NGM plates seeded with *Escherichia coli* OP50 at 20 °C.

Ot B was dissolved in DMSO for storage and diluted in PBS while in use. NGM plates containing various concentrations of Ot B were equilibrated overnight before use. The final DMSO concentration was kept at 0.1 % after with or without adding drugs to the plates.

### Lifespan Assay

Strains were cultured for 2-3 generations before using for lifespan analysis. Lifespan assay were conducted as described previously [15]. In brief, late L4 larvae or young adults were transferred to NGM plates containing inactivated OP50 (65 °C for 30 min) and 40 μM of 5-fluoro-2′-deoxyuridine (FUDR, Sigma) and scored every day. Animals were transferred to fresh plates with or without drugs every 2-4 days. Each experiment was repeated at least once. Means, SEM, and *P* value were shown in Table S1.

### Thermo-Tolerance and Body Movement Analysis

In thermo-tolerance assay, day 5 *C. elegans* were transferred to and maintained on plates with (50 μM) or without Ot B at 35 °C [39]. Live animals were scored the same way as in lifespan assays. At least 50 animals were used for each experiment. Statistical significance was determined by log-rank (Mantel-Cox) test.

Body movement assay was conducted as described previously [15, 18]. Briefly, for each experiment, at least 50 synchronized L4 larvae or young adult worms were treated and maintained as described in the lifespan assays. When tapping the plate, the animals moving in a continuous coordinated sinusoidal way were classified as fast movement.

### DAF-16:: GFP Localization Assay

DAF-16:: GFP localization assay were performed as described previously [40]. Briefly, 30 Synchronized young adult worms of transgenic strain TJ356 *daf*-*16(zls356) IV* [2] were transferred to the plates with (50 μM) or without Ot B, and cultured for 12-24 h at 20 °C, then monitored DAF-16::GFP signal by a fluorescent microscope system (Olympus, IX51).

### RNA Interference Experiments

In experiments before treatment, synchronized and grow to Late L4 larvae or young adults on NGM plates with OP50 were transferred to freshly-prepared NGM plates contained gene-specific RNAi bacteria. HT115 bacteria transformed with RNAi vectors (L4440) expressing dsRNA of the genes indicated were grown at 37 °C in LB with 100 µg mL^−1^ ampicillin, then seeded onto NGM plates containing 100 µg of 0.1 M IPTG.

### Quantitative RT-PCR Assay

Quantitative RT-PCR assay were carried out as described previously [15]. Briefly, Synchronized young adult wild-type worms cultured with (50 μM) or without Ot B at 20 °C for 24 h. Then, total RNA was extracted using RNAiso Plus (Takara) and converted to cDNA with High Capacity cDNA Reverse Transcription Kit (Applied Biosystems). The cDNA of *sod*-*3* was amplified and quantified in a Power SYBR Green PCR Master Mix (Applied Biosystems) on ABI 7500 DNA analyzer (Applied Biosystems). The experiments were conducted in triplicate and the results were carried out using 2^−△△CT^ method, and normalized to cdc-42.

## Electronic supplementary material

Supplementary material 1 (DOC 132 kb)
