# Supplementary material for: The Lifespan-Promoting Effect of Otophylloside B in Caenorhabditis elegans
Source: Nat Prod Bioprospect. 2015 Jun 26;5(4):177–83. doi: 10.1007/s13659-015-0064-4 (PMC4567989; doi:10.1007/s13659-015-0064-4)
Supplement: Supplementary file 1 — Supplementary material 1 (DOC 132 kb) [file 13659_2015_64_MOESM1_ESM.doc]

**The lifespan-promoting effect of otophylloside B in *Caenorhabditis elegans***

Jie Yang^a,b,c^, Qin-Li Wan^a,b^, Quan-Zhang Mu^a^, Chun-Feng Wu^a^, Ai-Jun Ding^a, b^, Zhong-Lin Yang^a, b^, Ming-Hua Qiu^a^, Huai-Rong Luo ^a, *^

^a^ State Key Laboratory of Phytochemistry and Plant Resources in West China, Kunming Institute of Botany, Chinese Academy of Sciences, Kunming, Yunnan 650201, China

^b^ University of Chinese Academy of Sciences, Beijing 100049, China

^c^ The Second Affiliated Hospital of Kunming Medical University, Kunming, 650101

Jie Yang and Qin-Li Wan authors contributed equally to this work.

*Corresponding author: Dr. Huai-Rong Luo

State Key Laboratory of Phytochemistry and Plant Resources in West China, Kunming Institute of Botany, Chinese Academy of Sciences

134 Lanhei Road, Kunming, Yunnan 650201, China

Phone: +86 871-65223225; Fax: +86 871-65223108

E-mail address: [luohuairong@mail.kib.ac.cn](mailto:luohuairong@mail.kib.ac.cn)

**Supplementary information**

**Contents:**

**Table S1. Effects of otophylloside B (Ot B) on lifespan**

**Table S2. Effects of otophylloside B (Ot B) on heat resistance**

**Table S3. Effects of otophylloside B (Ot B) on fast body movement**

**Table S1**

| **Figure** | **Strains** | **Treatments** | **Mean**  **Lifespan ± SEM**  **(days)** | ***P* value**  **VS**  **control** | **%**  **Change in mean lifespan** | **N** |
| --- | --- | --- | --- | --- | --- | --- |
|  | **N2 (WT)** |  |  |  |  |  |
| **1(B, C)** | EXP. 1 | 20℃/control | 21.667±0.204 |  |  | 168(5) |
|  | EXP. 1 | 20℃/ 20 µM Ot B | 23.103±0.313 | <0.0001 | 6.6 | 114(3) |
|  | EXP. 1 | 20℃/50 µM Ot B | 24.496±0.324 | <0.0001 | 13.1 | 121(4) |
|  | EXP. 1 | 20℃/200 µM Ot B | 23.667±0.204 | <0.001 | 7.2 | 111(3) |
|  | EXP. 1 | 20℃/500 µM Ot B | 22.086±0.236 | 0.253 | # | 116(2) |
|  | EXP. 2 | 20℃/control | 22.943±0.343 |  |  | 122 |
|  | EXP. 2 | 20℃/ 5 µM Ot B | 23.978690.252 | 0.02 | 4.3 | 139(2) |
|  | EXP. 2 | 20℃/ 20 µM Ot B | 24.400±0.339 | 0.017 | 6.3 | 120 |
|  | EXP. 2 | 20℃/200 µM Ot B | 24.692±0.318 | 0.001 | 7.6 | 120 |
|  | EXP. 2 | 20℃/500 µM Ot B | 23.041±0.285 | 0.563 | # | 123 |
|  | EXP. 3 | 20℃/control | 20.157±0.232 |  |  | 134 |
|  | EXP. 3 | 20℃/ 5 µM Ot B | 21.017690.323 | 0.02 | 4.3 | 115 |
|  | EXP. 3 | 20℃/ 20 µM Ot B | 21.548±0.233 | <0.0001 | 7 | 146 |
|  | EXP. 3 | 20℃/50 µM Ot B | 22.507±0.425 | <0.0001 | 11.6 | 69 |
|  | EXP. 3 | 20℃/200 µM Ot B | 20.94±0.306 | 0.001 | 4 | 117 |
|  | EXP. 3 | 20℃/500 µM Ot B | 20.991±0.344 | <0.0001 | 4.1 | 117 |
| **1(D)** | EXP. 1 | 20℃/control | 23.892±0.321 |  |  | 241 |
|  | EXP. 1 | 20℃/50 µM Ot B | 25.633±0.289 | 0.02 | 7.3 | 240 |
|  | EXP. 2 | 20℃/control | 21.667±0.204 |  |  | 166 |
|  | EXP. 2 | 20℃/50 µM Ot B | 24.538±0.334 | <0.0001 | 13.5 | 117 |
|  | ***daf-16(mu86)Ⅰ*** |  |  |  |  |  |
| **3(A)** | EXP. 1 | 20℃/control | 21.086±0.339 |  |  | 70(3) |
|  | EXP. 1 | 20℃/50 µM Ot B | 20.831±0.308 | 0.556 | # | 89(3) |
|  | EXP. 2 | 20℃/control | 20.824±0.378 |  |  | 85 |
|  | EXP. 2 | 20℃/50 µM Ot B | 20.278±0.313 | 0.11 | # | 108 |
|  | EXP. 3 | 20℃/control | 19.175±0.275 |  |  | 103 |
|  | EXP. 3 | 20℃/50 µM Ot B | 19.640±0.296 | 0.168 | # | 100 |
|  | ***sir-2.1(ok434)Ⅳ*** |  |  |  |  |  |
| **5(A)** | EXP. 1 | 20℃/control | 25.644±0.543 |  |  | 59(3) |
|  | EXP. 1 | 20℃/50 µM Ot B | 26.374±0.423 | 0.189 | # | 115(3) |
|  | EXP. 2 | 20℃/control | 20.441±0.188 |  |  | 49 |
|  | EXP. 2 | 20℃/50 µM Ot B | 20.796±0.373 | 0.238 | # | 102 |
|  | EXP. 3 | 20℃/control | 20.441±0.188 |  |  | 73 |
|  | EXP. 3 | 20℃/50 µM Ot B | 19.726±0.336 | 0.649 | # | 84 |
|  | ***eat-2(ad1116)Ⅱ*** |  |  |  |  |  |
| **5(B)** | EXP. 1 | 20℃/control | 23.000±0.246 |  |  | 107(2) |
|  | EXP. 1 | 20℃/50 µM Ot B | 28.000±0.582 | <0.0001 | 21.0 | 80(2) |
|  | EXP. 2 | 20℃/control | 20.229±0.356 |  |  | 83 |
|  | EXP. 2 | 20℃/50 µM Ot B | 21.922±0.317 | 0.001 | 8.3 | 103 |
|  | ***clk-1(e2519)Ⅲ*** |  |  |  |  |  |
| **5(C)** | EXP. 1 | 20℃/control | 28.253±0.382 |  |  | 83(2) |
|  | EXP. 1 | 20℃/50 µM Ot B | 28.596±0.616 | 0.109 | # | 57(2) |
|  | EXP. 2 | 20℃/control | 26.076±0.277 |  |  | 105 |
|  | EXP. 2 | 20℃/50 µM Ot B | 26.888±0.238 | 0.062 | # | 116 |
|  | ***skn-1(zu67)Ⅳ*** |  |  |  |  |  |
| **4(B)** | EXP. 1 | 20℃/control | 19.278±0.621 |  |  | 97(2) |
|  | EXP. 1 | 20℃/50 µM Ot B | 22.805±0.675 | <0.0001 | # | 128(2) |
|  | EXP. 2 | 20℃/control | 12.359±0.459 |  |  | 64 |
|  | EXP. 2 | 20℃/50 µM Ot B | 14.104±0.756 | 0.037 | # | 67 |
|  | EXP. 3 | 20℃/control | 22.609±0.584 |  |  | 92 |
|  | EXP. 3 | 20℃/50 µM Ot B | 23.970±0.700 | 0.008 | # | 67 |
|  | ***daf-2(e1370)Ⅲ*** |  |  |  |  |  |
| **4(A)** | EXP. 1 | 20℃/control | 38.433±0.443 |  |  | 104(3) |
|  | EXP. 1 | 20℃/50 µM Ot B | 37.874±0.443 | 0.486 | # | 103(3) |
|  | EXP. 2 | 20℃/control | 38.691±0.556 |  |  | 81 |
|  | EXP. 2 | 20℃/50 µM Ot B | 38.297±0.392 | 0.621 | # | 155 |
|  | EXP. 3 | 20℃/control | 37.609±0.579 |  |  | 115 |
|  | EXP. 3 | 20℃/50 µM Ot B | 38.491±0.485 | 0.728 | # | 114 |

The mean lifespan values were calculated by a logrank (Mantel-Cox) statistical test. *P* values were calculated for individual experiments, each consisting of control and experimental animals as the same time. “N” in the table shows the number of dead animals, with number of independent experiments in parentheses. All statistical were calculated by using SPSS package. “#”: no calculate (because *P*>0.05)

**Table S**2

| **Figure** | **Strains** | **Treatments** | **Mean Lifespan ± SEM**  **(hours)** | ***P* value**  **VS**  **control** | **%change in mean lifespan** | **N** |
| --- | --- | --- | --- | --- | --- | --- |
|  | **N2** |  |  |  |  |  |
| **2(B)** | EXP. 1 | 35℃/control | 11.829±0.315 |  |  | 117 |
|  | EXP. 1 | 35℃/50 µM Ot B | 14.219±0.361 | <0.0001 | 20.2 | 137 |
|  | EXP. 2 | 35℃/control | 9.573±0.333 |  |  | 164 |
|  | EXP. 2 | 35℃/50 µM Ot B | 14.949±0.342 | <0.0001 | 56.1 | 137 |
|  | EXP. 3 | 35℃/control | 9.139±0.363 |  |  | 165 |
|  | EXP. 3 | 35℃/50 µM Ot B | 13.888±0.347 | <0.0001 | 52.0 | 143 |

Lifespan experiments were carried out at 35℃. The mean lifespan and *P* values were calculated as described for Table S 1.

Table S3

| **Figure** | **Strains** | **Treatments** | **Fast body movement span (days)** | ***P* value**  **VS**  **control** | **N** |
| --- | --- | --- | --- | --- | --- |
|  | **N2** |  |  |  |  |
| **2(A)** | EXP. 1 | 20℃/control | 13.500±0.086 |  | 82 |
|  | EXP. 1 | 20℃/50 µM Ot B | 14.507±0.184 | <0.0001 | 73 |
|  | EXP. 2 | 20℃/control | 14.878±0.1818 |  | 98 |
|  | EXP. 2 | 20℃/50 µM Ot B | 16.600±0.183 | <0.0001 | 120 |
|  | EXP. 3 | 20℃/control | 13.359±0.185 |  | 92 |
|  | EXP. 3 | 20℃/50 µM Ot B | 14.635±0.177 | <0.0001 | 115 |

Fast body movement experiments were carried out at 20℃. All statistical were calculated by using SPSS package. The log rank (Mantel-Cox) test was used for statistical analysis. In each experiment, control and experimental animals were analyzed in parallel. N: total number of analysis.
